# Vitamin D assessment in patients with COVID-19 virus and correlation with severity

**DOI:** 10.1186/s43162-022-00142-8

**Published:** 2022-07-08

**Authors:** Tarek M. Yosef, Shereen A. Saleh, Sara Fekry Ali, Ahmed Elmetwally Ahmed

**Affiliations:** Gastroenterology, Hepatology and Internal Medicine Department, Ain Shams University, Cairo, Egypt

**Keywords:** Vitamin D, COVID-19 virus, Neutrophil-lymphocyte ratio

## Abstract

**Background:**

Vitamin D may play a vital role in preventing the multi-system consequences of COVID-19 infections. The aim of this study is to evaluate the potential association between mean serum levels of vitamin D and COVID-19 and its correlation with severity and mortality.

**Results:**

A case-control study conducted on 80 Egyptian patients admitted at Ain Shams University designated hospitals, Cairo, Egypt, from March 2021 to September 2021. Regarding the laboratory investigations, we found that COVID-19 cases have significantly lower lymphocytic counts than controls. Regarding vitamin D, this study showed a statistically significant positive correlation between vitamin D and lymphocytes, and there were statistically significant negative correlations between vitamin D, neutrophil-lymphocyte ratio, C-reactive protein, blood urea nitrogen, serum creatinine, aspartate aminotransferase, alanine aminotransferase, ferritin, lactate dehydrogenase, and D-dimer.

**Conclusion:**

This study confirms that vitamin D deficiency is associated with the severity of COVID-19 clinically and laboratory.

## Background

The role of vitamin D in COVID-19 is not well known since the virus pathophysiology is still new to us. But there is a reason sounding behind this speculation [1].

T regulatory lymphocytes (TRegs) are the main defense mechanism against uncontrolled inflammation. The number of TRegs was found to be markedly lower in patients with severe COVID-19 infection while increased levels were associated with a reduced level of severe respiratory disease. TReg levels can be increased by vitamin D intake. Also, low serum vitamin D concentrations are usually associated with a rise in inflammatory cytokines which raises the possibility that normal serum vitamin D concentration may reduce the incidence of cytokine storm which can occur in severe COVID-19 infection [2].

Vitamin D deficiency is an important risk factor for infections due to insufficient innate immune system reactivity, so, testing of serum vitamin D at the wintertime (as it has usually the lowest levels expected) should be mandatory to be done as part of regular health check-up status. The test is more vital especially in patients with chronic illnesses, where we should expect vitamin D insufficiency throughout the year with an increased burden of disease severity up to inevitable mortality [2].

Also, thrombotic complications especially in the pulmonary microvasculature are common in COVID-19 and vitamin D deficiency is also associated with a rise in thrombotic events which increases further speculation about the significance of adequate serum vitamin D concentration in COVID-19 [2].

The aim of this study is to assess if there is a potential association between mean serum levels of vitamin D and COVID-19 and its correlation with the severity of COVID-19.

## Methods

This was a case-control study conducted at Ain Shams University designated hospitals from March 2021 to September 2021. The study population included 80 adult participants ≥18 years. All patients underwent a real-time reverse transcriptase-polymerase chain reaction (RT-PCR) assay for COVID-19 for nasopharyngeal and oropharyngeal swab specimens.

An informed written consent was obtained from all patients before inclusion in this study. The study was approved by the Ethical Review Board of our institution **(**M S 742 /2020/ 2021**)**. The study protocol conforms to the ethical guidelines of the Declaration of Helsinki.

The participants were divided into two groups: Group I (control): 20 apparently healthy subjects who were previously on vitamin D supplementation and stopped treatment 3 months before enrollment in the study. Group II (patients): 60 COVID-19-positive patients that were further divided into three groups according to the severity of the disease. Group IIA: 20 mild symptomatic COVID-19-positive patients. Group IIB: 20 moderate to severe symptomatic COVID-19 positive patients. Group IIC: 20 critically ill COVID-19-positive patients.

COVID-19 severity was classified as mild, moderate, severe, or critical according to World Health Organization (WHO) guidelines. Mild cases are symptomatic patients without evidence of viral pneumonia or hypoxia. Moderate cases are patients with clinical signs of pneumonia and pneumonic manifestations that can be seen in chest imaging but no signs of severe pneumonia including SpO2 ≥90% on room air. Severe cases include patients with clinical signs of pneumonia and one of the following: respiratory rate >30 breaths/minute, severe respiratory distress, or SpO2 <90% on room air. Critical cases are patients meeting any of the following criteria: occurrence of respiratory failure requiring mechanical ventilation, presence of septic shock, and multi-organ failure that requires monitoring and treatment in the intensive care unit (ICU).

### Exclusion criteria

Exclusion criteria include patients with dementia, patients with learning disability, patients with mental health needs and alcohol or drug dependency, pregnant women, and patients with sarcoidosis, hypercalcemia, and known vitamin D intolerance.

All patients were subjected to the following: full history taking, full clinical examination, laboratory investigations that included: PCR for oropharyngeal and nasopharyngeal swab specimens, complete blood count [white blood cell count (WBC), lymphocytic count, hemoglobin and platelet count], neutrophil-lymphocyte ratio (NLR), C-reactive protein (CRP), erythrocyte sedimentation rate (ESR), kidney function tests [serum creatinine, blood urea nitrogen (BUN), and uric acid], liver function tests and enzymes [alanine aminotransferase (ALT), aspartate aminotransferase (AST), total/direct bilirubin and serum albumin], ferritin, lactate dehydrogenase (LDH), D-Dimer, and Vit D (25 hydroxy cholecalciferol) by ELISA. High resolution computed tomography (HRCT) chest was done to determine the COVID-19 Reporting and Data System (CORADs).

### Statistical analysis

Data were collected, revised, coded, and entered to the Statistical Package for Social Science (IBM SPSS) version 20. The qualitative data were presented as numbers and percentages while quantitative data were presented as mean, standard deviations, and ranges when their distribution was found parametric.

The comparison between two groups with qualitative data was done by using chi-square test and/or Fisher exact test was used instead of chi-square test when the expected count in any cell was found less than 5.

The comparison between two independent groups with quantitative data and parametric distribution was done by using independent *t*-test.

The confidence interval was set to 95% and the margin of error accepted was set to 5%. So, the p-value was considered significant as the following: *P*> 0.05 = non-significant (NS), *P* < 0.05 = significant (S), and *P* < 0.001 = highly significant (HS).

## Results

Results are shown in Tables 1, 2, 3, and 4.

**Table 1:** Demographic data of patient groups with mild, moderate, and severe COVID-19 manifestations and control subjects

		Mild ***n***=20	Moderate ***n***=20	Severe ***n***=20	Control ***n***=20	***P*** value
**Age (years)** **(Mean ±SD)**		55.400 ±16.791	59.550 ±13.953	62.750 ±15.003	25.700 ±3.197	<0.001
**Sex (n, %)**	Male	13 (65%)	9 (45%)	13 (65%)	11 (55%)	0.522
	Female	7 (35%)	11 (55%)	7 (35%)	9 (45%)	
**Smoking (*n*, %)**	Smokers	13 (65%)	16 (80%)	10 (50%)	18 (90%)	0.030
	Non-smokers	7 (35%)	4 (20%)	10 (50%)	2 (10%)	
**Medical history (*n*, %)**	Free	9 (45%)	3 (15%)	5 (25%)	20 (100%)	<0.001
	HTN	6 (30%)	6 (30%)	6 (30%)	0	
	DM	2 (10%)	5 (25%)	0	0	
	AF+HTN	0	2 (10%)	1 (5%)	0	
	DM+HTN	3 (15%)	4 (20%)	8 (40%)	0	

*P* value >0.05: non-significant (NS), *P* value <0.05: significant (S), *P* value< 0.01: highly significant (HS)

**Table 2:** Laboratory and radiologic findings of COVID-19 patient groups: mild, moderate, and severe and control subjects

		Mild ***n***=20	Moderate ***n***=20	Severe ***n***=20	Control ***n***=20	***P*** value
**TLC (10**^**9**^**/L)** **(Mean ±SD)** **(range)**		5.24±2.029 2.3–10.2	12.7±6.56 4.3–32.5	12.3±7.8 3.3–33	7.24±1.84 4.5–10.2	<0.001
**Lymphocytes (10**^**9**^**/L)** **(Mean ±SD)** **(range)**		0.85±0.344 0.5–2	0.77±.533 0.3–2	0.48±0.337 0.2–1.4	2.010±0.799 1–3	<0.001
**Neutrophils** **(10**^**9**^**/L)** **(Mean ±SD)** **(range)**		4.07±2.05 1–9	11.085±6.337 3.7–30.7	10.87±7.48 2.2–31.9	4.830±1.913 2–8	<0.001
**NLR** **(Mean ±SD)** **(range)**		5.25±3.127 1–13	20.6±13.59 2–43	31.90±26.80 2–106	2.8±1.47 1–6	<0.001
**Hemoglobin (g/dl)** **(Mean ±SD)** **(range)**		12.9 ±2.14 10–18.5	11.78 ±2.555 6.5–15	11.96 ±2.491 7–18	12.185 ±1.267 10–14.5	0.385
**Platelet (10**^**9**^**/L)** **(Mean ±SD)** **(range)**		5.25 ±3.127 1–13	20.65 ±13.59 2–43	31.9 ±26.8 2–106	2.8 ±1.47 1–6	<0.001
**CRP (mg/dl)** **(Mean ±SD)** **(range)**		24.5 ±19.8 0.1–62	30.95 ±26.95 11.28–100	78.99 ±68.10 10.7–230	4.15 ±2.09 0.5–6	<0.001
**ESR (mm/hr)** **(Mean ±SD)** **(range)**		24.8 ±17.8 5.1–70	26.55 ±23.23 10–80	44.01 ±28.17 10–90	3.67 ±2.34 0.5–6	<0.001
**BUN (mg/dl)** **(Mean ±SD)** **(range)**		14.8 ±5.6 8–29	41.1 ±21.59 15–88	53.45 ±28.34 17–105	10.0 ±3.62 5–17	<0.001
**S.creatinine (mg/dl)** **(Mean ±SD)** **(range)**		0.86 ±0.34 0.3–1.9	1.03 ±0.41 0.4–2.1	1.5 ±0.81 0.5–3.1	0.82 ±0.30 0.4–1.5	<0.001
**AST (IU/L)** **(Mean ±SD)** **(range)**		24.6 ±6.41 15–42	53.05 ±47.5 9–215	72.0 ±58.44 4–212	14.85 ±6.029 5–25	<0.001
**ALT (IU/L)** **(Mean ±SD)** **(range)**		31.6 ±13.9 11–69	37.9 ±30.19 9–129	49.75 ±38.09 13–138	14.9 ±7.33 7–40	0.001
**Total bilirubin (mg/dl)** **(Mean ±SD)** **(range)**		0.87 ±0.29 0.4–1.5	1.02 ±0.265 0.6–1.6	0.84 ±0.35 0.4–1.6	0.69 ±0.22 0.3–1	0.006
**S.albumin (g/dl)** **(Mean ±SD)** **(range)**		3.43±0.26 3.1–3.9	3.21±0.59 1.8–4.2	3.02±0.54 2.1–3.8	3.66±0.26 3.5–4.5	<0.001
**Ferritin (ng/ml)** **(Mean ±SD) (range)**		245.6±135.5 56–677.2	589.6±368.3 112.5–1434	683.4±556.6 26.7–2100	73.0±48.96 20–210	<0.001
**LDH (IU/L)** **(Mean ±SD)** **(range)**		256.4±73.1 110–350	487.5±303.2 125–963	603±239 225–986	160±19.47 140+200	<0.001
**D-dimer (mg/L)** **(Mean ±SD) (range)**		0.45±0.19 0.2–0.9	1.16±0.97 0.212–2.9	2.2±1.755 0.3–6.9	0.309±0.08 0.1–0.45	<0.001
**Vitamin D (ng/ml)** **(Mean ±SD)** **(range)**		24.00±3.7 17–30	21.5±3.95 11–27	16.6±4.16 7–23	56.65±14.9 35–89	<0.001
**CORADS** **(*n*, %)**	**1**	0	0	0	20 (100%)	<0.001
	**3**	15 (75%)	10 (50%)	3 (15%)	0	
	**4**	3 (15%)	6 (30%)	5 (25%)	0	
	**5**	2 (10%)	4 (20%)	12 (60%)	0	

*P* value >0.05: non-significant (NS), *P* value <0.05: significant (S), *P* value < 0.01: highly significant (HS)

**Table 3:** Relation between vitamin D and sex, special habits, medical history ,and CORADS classification

		Vitamin D (ng/ml)		***T***-test or ANOVA	
		*N*	Mean ± SD	*T* or *F*	*P* value
**Sex**	**Male**	35	20.000±5.358	−1.295	0.200₰
	**Female**	25	21.680±4.318		
**Smoking status**	**Non-smoker**	39	21.667±4.664	2.107	0.039₰
	**Smoker**	21	18.905±5.166		
**Medical history**	**Free**	17	20.176±4.694	1.794	0.143•
	**HTN**	18	19.944±5.557		
	**DM**	7	23.571±2.507		
	**AF+HTN**	3	15.667±6.429		
	**HTN+DM**	15	21.867±4.612		
**CORADS classification**	**CORADS 3**	28	24.607±2.699	71.693	<0.001•
	**CORADS 4**	14	20.429±1.453		
	**CORADS 5**	18	14.833±3.365		

*P* value >0.05: non-significant (NS), *P* value <0.05: significant (S), *P* value< 0.01: highly significant (HS)

₰Independent *t*-test

•One-way ANOVA

**Table 4:** Correlation of vitamin D and age, different laboratory, and radiologic findings of COVID-19 patients

Correlations		
	**Vitamin D**	
	*R*	*P* value
**Age (years)**	− 0.117	0.374
**TLC (10**^**9**^**/L)**	− 0.194	0.138
**Lymphocytes (10**^**9**^**/L)**	0.270	0.037*
**Neutrophils (10**^**9**^**/L)**	− 0.182	0.164
**Hemoglobin (g/dl)**	0.124	0.344
**Platelets (10**^**9**^**/L)**	− 0.044	0.737
**Neutrophil-lymphocyte ratio**	− 0.295	0.022*
**CRP (mg/dl)**	− 0.259	0.046*
**ESR (mm/h)**	− 0.167	0.201
**BUN (mg/dl)**	− 0.315	0.014*
**Serum creatinine (mg/dl)**	− 0.256	0.048*
**AST (IU/L)**	− 0.412	0.001*
**ALT (IU/L)**	− 0.337	0.008*
**Total bilirubin (mg/dl)**	0.037	0.778
**S. albumin (g/dl)**	0.248	0.056
**Ferritin (ng/ml)**	− 0.463	<0.001*
**LDH (IU/L)**	− 0.269	0.038*
**D-dimer (mg/L)**	− 0.382	0.003*

*P value <0.05: significant, *P* value <0.01: highly significant

**Fig. 1:**
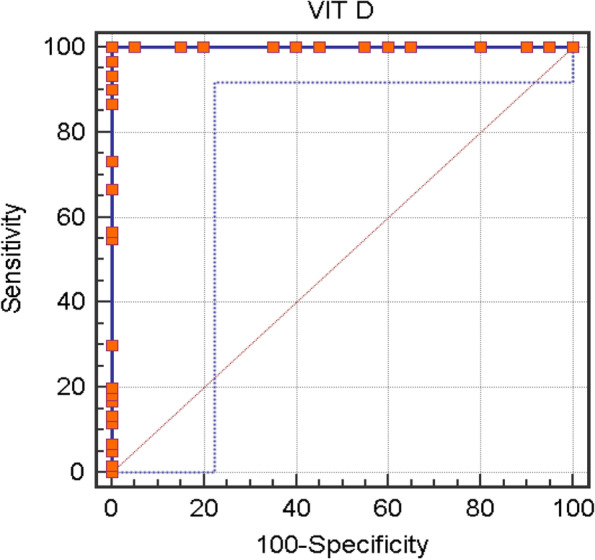
ROC curve for diagnostic performance of vitamin D in detecting COVID-19 patients

Receiver operating characteristic (ROC) curve was plotted to identify the best cut-off value of vitamin D to predict COVID-19 cases. As shown in Table 5 and Fig. 1, vitamin D at cut-off value ≤ 30 ng/ml have a sensitivity and specificity of 100%.

**Table 5:** Diagnostic performance of vitamin D in detecting COVID-19 patients

	Cut-off	Sensitivity	Specificity	PPV	NPV	Accuracy
Vitamin D	≤30 ng/ml	100%	100%	100%	100%	100%

## Discussion

Vitamin D insufficiency had been found to contribute to acute respiratory distress syndrome, the main cause of death caused by COVID-19. Vitamin D plays a vital role in strengthening the body’s immunity by stimulating monocyte differentiation and suppressing lymphocyte proliferation [3].

The current study compared vitamin D serum levels with different health-related scores, inflammatory markers, markers of cellular damage and coagulation, and radiological findings during COVID-19 illness. Interestingly, patients with low vitamin D plasma levels had compromised biochemical and clinical findings reflected by the profound immunological involvement. Vitamin D insufficiency and deficiency were common in COVID-19-affected patients.

Regarding the laboratory investigations, [4] found that LDH had increased significantly in most patients, while albumin had decreased, but ALT and AST showed no significant changes. Another study by [5] showed that 2–11% of patients with COVID-19 had liver comorbidities and 14–53% of cases had abnormal ALT and AST levels during the progression of COVID-19 disease which supports our results.

Also, Shi et al. [6] found that incidence of AST abnormality among the patients diagnosed by CT chest was significantly lower than those diagnosed after the onset of symptoms. Therefore, liver injury is more common in severe cases as compared to mild cases of COVID-19. On the other hand, Yang et al. [7] found no significant difference in the liver function between survivors (30%) and non-survivors (28%). Liver injury in mild cases of COVID-19 is usually transient and can return back to normal or its baseline without any special treatment [5].

We had found that the number and percentage of WBC, lymphocytes, and neutrophils were significantly different between positive COVID-19 cases and the control group. In comparison to the normal range, we found low lymphocytic counts in patients with positive COVID-19, whereas neutrophil counts were higher in these patients and this agrees with [8] study.

Viral particles usually spread throughout the respiratory tract and infect other cells stimulating a series of immune responses and causing changes in the white blood cells especially lymphocytes. Some studies suggest that a significant decrease in the number of lymphocytes suggests that the coronavirus affects the immune cells and suppresses the cellular immune function [4]. Tsui et al. [9] reported that increased neutrophil count and elevated LDH levels were independent predictors of an adverse clinical outcome.

Our study showed a statistically significant positive correlation between vitamin D and lymphocytes, and there was a statistically significant negative correlation between vit D, neutrophil-lymphocyte ratio, CRP, BUN, serum creatinine, AST, ALT, ferritin, LDH, and D-dimer.

Meltzer et al. [10] in a retrospective cohort study done at the University of Chicago showed that the rate of COVID-19 was higher in a vitamin D-deficient group than a vitamin D-sufficient group.

Also, [11] in a retrospective cohort study done in Swiss patients showed significantly lower levels of vitamin D in COVID-positive patients (median value 11.1 ng/mL) as compared with negative patients (24.6 ng/mL).

A study done by Kara et al. [12] showed that vitamin D deficiency was a pandemic especially in Europe, where 40% were vitamin D-deficient and 13% severely deficient.

Mendy et al. [13] found that the severity of COVID-19 disease was associated with vitamin D deficiency and the likelihood of ICU admission were higher among vitamin D–deficient patients.

Daneshkhah et al. [14] found that treating severe vitamin D deficiency decreased the risk of increased CRP levels which are used as a marker of cytokine storm, which usually occur in severe COVID-19 cases.

Lau et al. [15] revealed that eleven (84.6%) ICU patients that were respiratory compromised due to COVID-19 and four (57.1%) non-ICU patients had vitamin D deficiency. ICU patients included ten patients with hypertension and six patients with diabetes mellitus. Non-ICU patients included five hypertensive and one diabetic patient. Of these, 64.6% (*n*=7) had low vitamin D levels (<20 μg/mL) and 3% had critically low levels (<10 μg/mL)

One trial by Grazon [16] showed that intake of a single oral dose of 25,000 IU (625 μg) of vitamin D would improve mortality in patients with COVID-19.

Castillo et al. [17] showed that high doses of calcifediol reduce ICU admission for hospitalized patients.

## Conclusion

In conclusion, this study confirms that vitamin D deficiency is associated with the severity of COVID-19 clinically and laboratory.

## Data Availability

The datasets used and analyzed during the current study are available from the corresponding author upon reasonable request.
